# Single femoral artery access is safe and feasible during transcatheter aortic valve replacement: a propensity score matched analysis

**DOI:** 10.3389/fcvm.2023.1228258

**Published:** 2023-11-03

**Authors:** Yunfeng Yan, Jing Yao, Fei Yuan, Xinmin Liu, Taiyang Luo, Zhinan Lu, Sanshuai Chang, Qian Zhang, Ran Liu, Chengqian Yin, Guangyuan Song

**Affiliations:** Beijing Institute of Heart Lung and Blood Vessel Disease, Beijing Anzhen Hospital, Capital Medical University, Beijing, China

**Keywords:** transcatheter aortic valve replacement, simplification, single artery access procedure, propensity matched analysis, transfemoral

## Abstract

**Background:**

Transcatheter aortic valve replacement (TAVR) potentially may be significantly simplified by using the single artery access (SA) technique, which does not require a secondary artery access. Nevertheless, the safety and efficacy of this technique remains unclear. Our goal was to determine if single artery access TAVR (without upgrading the sheath size) is a feasible, minimally invasive procedure.

**Methods:**

Patients with symptomatic severe aortic stenosis who underwent TAVR via the femoral artery were consecutively enrolled in this study. Eligible individuals were divided into 2 groups: the SA group and the dual artery access (DA) group. The primary end point was device success (defined by the valve academic research consortium 3, VARC 3). A 6-month follow-up and propensity score matching analyses were performed.

**Results:**

After propensity score matching analysis, a total of 130 patients were included: 65 in the SA group and 65 in the DA group. The SA procedure achieved similar device success (95.4% vs. 87.7%; *P *= 0.115) compared with the DA procedure. The SA procedure shortened the operating time (102 min vs. 125 min; *P *= 0.001) but did not increase the x-ray time or dose. Both a 20 Fr and a 22 Fr sheath (without upgrading the sheath size) could be used for the SA procedure. There was no major vascular complication occurred in both groups. The incidence of minor main vascular and access complications in the SA group was comparable to those of the DA procedure (0.0% vs. 3.1%; *P *= 0.156).

**Conclusions:**

The SA access procedure is a promising minimally invasive TAVR technique with a low incidence of vascular complications and a high incidence of device success. It is safe and possibly applicable in all TAVR procedures.

## Introduction

Since the first aortic valve prosthesis percutaneously implanted in 2002 (1), the evolution of transcatheter aortic valve replacement (TAVR) has been astonishing. Currently, TAVR is recommended for treating symptomatic severe aortic stenosis (AS) regardless of the surgical risk (2–4). The feasibility of a simplified TAVR has been explored extensively and is regarded as another revolution in the development of this technique (5).

As major vascular complications of approximately 10% are reported from current all-comers cohorts (6, 7), improvements in vascular access are required to improve the outcome of TAVR. In contrast to the fact that the transfemoral approach is recommended as the first choice for primary access (3, 8), the choice of secondary access is widely debated. Conventionally, the contralateral femoral artery has been the first choice. However, as many as 25% of all vascular/access complications are associated with a transfemoral secondary access (9, 10). A radial artery access can be applied and has been proven to be a useful alternative that results in fewer complications (9, 11, 12). Previous studies have also proven the safety of the unilateral femoral access (13). However, secondary access complications still occurred.

Previous studies have explored the possibility of eliminating secondary access. Some researchers compared the effects of puncturing only 1 femoral artery as the primary access with the effects of conventional dual artery (DA) access and reported promising results (14). However, they used aortic root calcifications as landmarks to avoid the use of angiography, which requires rigorous patient selection and is rarely used. Other researchers who conducted a 1-arm study reported that they placed both the delivery system and the pigtail into the sheath, thereby achieving promising outcomes (15).

In the present study, we analyzed our modified TAVR process with a single femoral artery puncture and assessed whether this technique was safer and more effective than the DA procedure.

## Methods

### Study design and patients

This trial was an all-comer, single-center, retrospective cohort study that was performed at the Beijing Anzhen Hospital between June 2021 and September 2022. The protocol was approved by the clinical research ethics committee of Beijing AnZhen Hospital (No.: 2021008X). An adjudication board blinded to the patient groups was responsible for enrollment, judgment about end points, quality control, and other related issues.

All individuals with symptomatic severe AS who had TAVR via the femoral artery were consecutively enrolled in this study. The inclusion criteria comprised (1) patients with severe AS; (2) patients with symptoms associated with AS: dyspnea related to heart failure (New York Heart Association Functional class ≥II), angina, syncope/presyncope, and others; (3) aortic root anatomy that was suitable for TAVR that was assessed by contrast-enhanced multidetector computerized tomography; (4) patients ≥60 years old (4); and (5) suitable iliofemoral access diameter assessed by contrast-enhanced multidetector computerized tomography.

The exclusion criteria included (1) a myocardial infarction within ≤1 month; (2) hypertrophic cardiomyopathy with obstruction; (3) evidence of an intracardiac mass, thrombus, or vegetation; (4) inability to tolerate antithrombotic/anticoagulation therapy; (5) evidence of septicemia or endocarditis; (6) life expectancy <12 months; (7) significant aortic or other diseases requiring surgical intervention: aortic dissection, aortic aneurysm (especially if >5 cm); (8) the use of a sheath or a transcatheter heart valve (THV) delivery system did not permit the insertion of 2 instruments [for example, an e-sheath (Edwards Lifesciences, Irvine, CA, USA)].

### Grouping

Based on the SA technology applied, eligible patients were assigned to 1 of 2 groups: the SA group or the conventional DA group. That is: patients were assigned to different groups according to their final treatment strategy.

### Single artery access procedure

**Figure 1:**
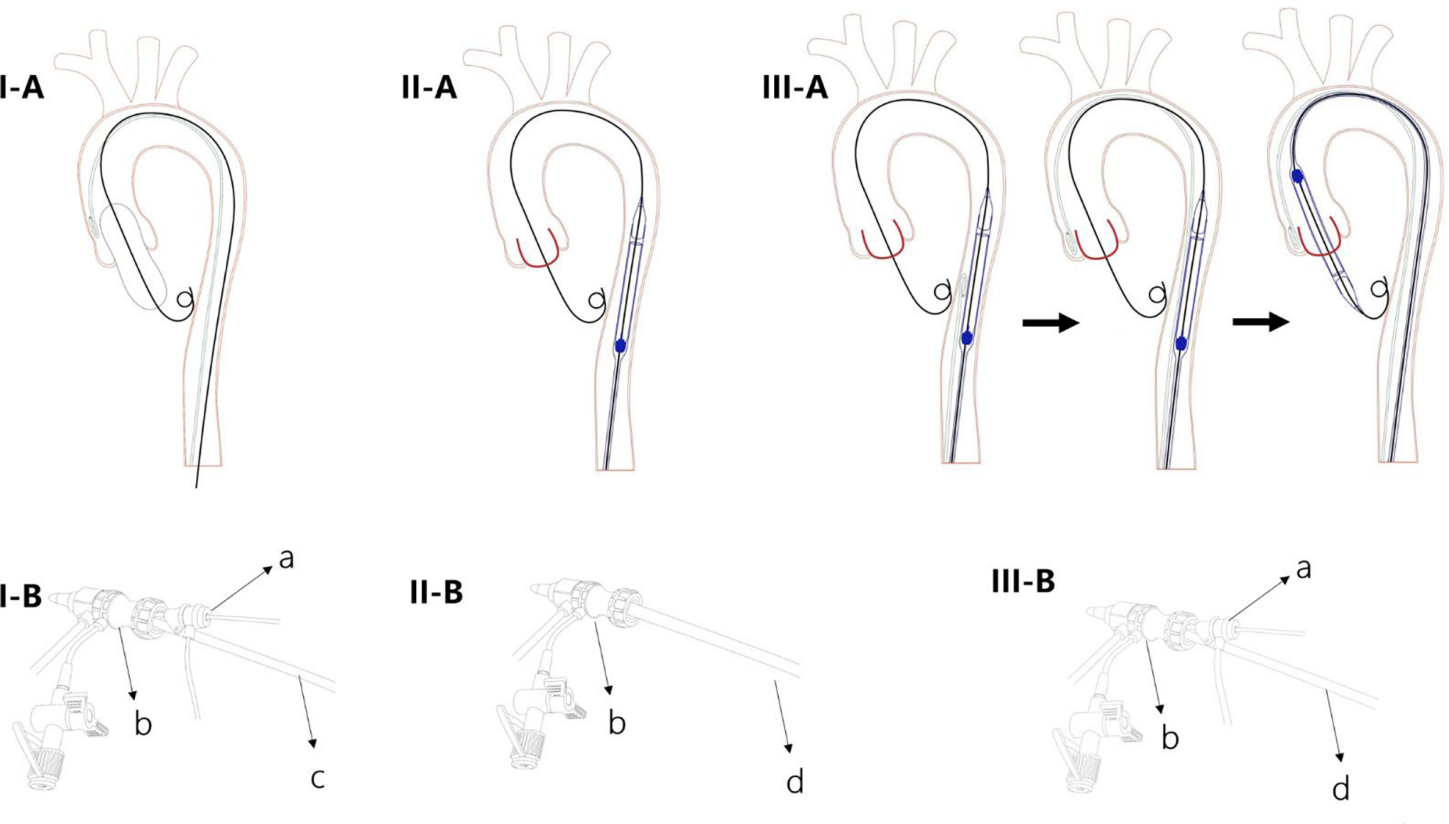
Diagram of the single artery access technique. I (**A,B**) A 5 Fr pigtail catheter is inserted into the aortic root for an aortogram with the 5 Fr sheath inside the large sheath; balloon dilation occurs through the same sheath. II (**A,B**) After the 5 Fr pigtail and the balloon are removed, the transcatheter heart valve (THV) delivery system is inserted into the descending aorta. III (**A,B**) The pigtail catheter is inserted from the single femoral access up to the bottom of the aortic root; then the THV is delivered to the aortic root. (**a**) A 5F sheath with pigtail catheter; (**b**) large sheath; (**c**) balloon; (**d**) THV delivery system.

The key point of the SA procedure is to avoid using a secondary artery (without upgrading the sheath size). A brief description of the SA procedure is provided below (Figure 1):

1. Establish femoral access: perform an ultrasound-guided precision puncture, preset 2 Perclose ProGlide devices (Abbott Laboratory, Chicago, IL, USA), and insert the sheath.
2. Aortogram: insert a pigtail catheter into the aortic root for the aortogram.
3. Balloon predilation: After predilating the balloon, remove the pigtail catheter.
4. Delivery of the THV: (i) Insert the THV delivery system into the descending aorta; (ii) insert the pigtail catheter from the single femoral access point to the bottom of aortic root; and (iii) deliver the THV to the aortic root.
5. Deployment of the THV: Using the pigtail catheter and the aortogram as guides, release the THV.
6. Removal of the sheath and suturing: Inject the contrast agent slowly while removing the sheath to detect potential vascular injury. Use 2 Perclose ProGlide devices to lock the puncture orifice.

It should also be noted that rapid cardiac pacing is performed through either the left ventricular wire or a preinstalled temporary pacemaker wire via the femoral vein.

### Dual artery access procedure

Traditionally, two arteries are punctured: the primary access used for valve delivery and the secondary access for aortic root aortogram. That is the DA access procedural.

### Data extraction

The following data were extracted from the hospital information system by trained researchers: baseline clinical history (such as: age, sex, hypertension, and dyslipidemia), examination and laboratory information, medical information, and data on endpoints.

### Definition

Severe AS was defined according to transthoracic echocardiographic results: (1) jet velocity ≥400 cm/s or mean gradient ≥40 mmHg; and/or (2) aortic valve area ≤1.0 cm^2^ or aortic valve area index ≤0.6 cm^2^/m^2^ (2–4).

### Procedural and clinical end points

The primary end point was device success, which was defined according to the Valve Academic Research Consortium 3 (VARC−3) criteria: a composite of technical success, freedom from mortality, freedom from surgery or intervention related to the device/ complications, and intended performance of the valve during 30 days follow-up (16). Secondary end points included death; cardiac death; major main vascular/access-related complications; minor vascular/access-related complications; major secondary vascular/access-related complications; minor secondary vascular/access-related complications; myocardial infarction; acute kidney injury; moderate/severe paravalvular regurgitation; new permanent pacemaker; new left bundle branch block; rehospitalization; and disabling stroke.

The following procedural data were also extracted: operating time, x-ray time, x-ray dose, concurrent percutaneous transluminal coronary intervention, valve-in-valve implant, sheath size, and bioprosthetic valve type. The definition of operating duration was the time from the introduction of anesthesia until the patient left the operating room. x-ray time and dose were collected directly by a digital subtraction angiography device.

### Follow-up procedures

The follow-up procedures were performed by trained researchers who were blinded to the patients’ groups. Data were collected at 1 month and 6 months during clinical visits and by phone calls and the We-chat application.

### Statistical analyses

The continuous variables with normal distributions were presented as the means ± standard deviations and were compared using the Student *t*-test or the analysis of variance test. Those variables without normal distributions were presented as medians with interquartile ranges, and differences between the groups were analyzed using the Mann–Whitney *U*-test or the Kruskal–Wallis test. The categorical data were presented as numbers and percentages and were compared using the *χ*^2^ test or the Fisher exact test, where applicable.

All end points were presented as numbers and percentages and were analyzed by using the *χ*^2^ test or the Fisher exact test, where applicable.

To balance selection biases and other confounding items, a propensity score matched analysis was performed, based on the patients’ baseline characteristics, which are listed in Table 1. We completed a nonparsimonious model. Patients were matched 1:1 using a nearest-neighbor algorithm, with a caliper of 0.05. The absolute standardized differences were used to evaluate the imbalance of each variable. The propensity score matched baseline characteristics of the patients were then compared again using the Student *t*-test, a Wilcoxon rank-sum test, a *χ*^2^ test, or the Fisher exact test, where applicable. The end points of the propensity score matched population were analyzed using the *χ*^2^ test or the Fisher exact text, where applicable.

**Table 1:** Baseline characteristics.

	Total population (*n* = 213)			Propensity matched population (*n* = 130)		
	SA (*n* = 113)	DA (*n* = 100)	*P* Value	SA (*n* = 65)	DA (*n* = 65)	*P* Value
Age (years)	73.59 ± 8.59	74.39 ± 7.73	0.480	73.38 ± 9.15	73.40 ± 7.04	0.991
Male	57 (50.4)	62 (62.0)	0.090	35 (53.8)	42 (64.6)	0.212
Hypertension	69 (61.1)	59 (59.0)	0.759	36 (55.4)	39 (60)	0.594
Diabetes	36 (31.9)	24 (24.0)	0.203	16 (24.6)	18 (27.7)	0.690
Coronary artery disease	30 (26.5)	21 (21.0)	0.344	14 (21.5)	16 (24.6)	0.677
Prior MI	2 (1.8)	2 (2.0)	0.902	2 (3.1)	2 (3.1)	1.000
Prior PCI	16 (14.2)	12 (12.0)	0.642	8 (12.3)	9 (13.8)	0.795
Prior CABG	2 (1.8)	1 (1.0)	0.635	0 (0.0)	1 (1.5)	0.317
Prior stoke	5 (4.4)	12 (12.0)	**0**.**042**	5 (7.7)	5 (7.7)	1.000
PVD	11 (9.7)	10 (10.0)	0.948	7 (10.8)	8 (12.3)	0.784
Dyslipidemia	98 (86.7)	80 (80.0)	0.186	53 (81.5)	57 (87.7)	0.331
COPD/asthma	9 (8.0)	9 (9.0)	0.786	4 (6.2)	5 (7.7)	0.730
CKD	9 (8.0)	6 (6.0)	0.576	5 (7.7)	5 (7.7)	1.000
Atrial fibrillation/flutter	12 (10.6)	11 (11.0)	0.929	6 (9.2)	8 (12.3)	0.571
Permanent pacemaker	3 (2.7)	0 (0.0)	0.102	0 (0)	0 (0)	NA
Left bundle branch block	7 (6.2)	7 (7.0)	0.813	6 (9.2)	5 (7.7)	0.753
Right bundle branch block	14 (12.4)	2 (2.0)	**0**.**004**	3 (4.6)	1 (1.5)	0.312
NYHA III or IV	75 (66.4)	65 (65.0)	0.833	42 (64.6)	41 (63.1)	0.855
Smoking	25 (22.1)	24 (24.0)	0.745	15 (23.1)	17 (26.2)	0.684
Drinking BMI (kg/m^2^)	16 (14.2)	13 (13.0)	0.806	8 (12.3)	9 (13.8)	0.795
	24.47 ± 3.09	24.32 ± 3.27	0.720	23.92 ± 2.76	24.80 ± 3.19	0.096
BSA	1.79 ± 0.15	1.81 ± 0.18	0.494	1.79 ± 0.15	1.82 ± 0.18	0.195
EuroSCORE II	2.40 (1.64–4.20)	2.37 (1.55–4.85)	0.637	2.21 (1.51–3.92)	2.17 (1.37–4.04)	0.524
UCG information						
LVEF (%)	58.00 (43.00–64.00)	58.00 (46.00–62.00)	0.883	60.00 (54.00–65.00)	58.00 (46.00–63.75)	0.466
LVESD	34.00 (29.00–46.00)	33.00 (29.00–45.00)	0.769	33.00 (28.00–44.00)	34.50 (28.25–46.00)	0.336
LVEDD	51.00 (46.00–58.00)	51.00 (46.00–61.00)	0.721	50.00 (46.00–57.00)	51.50 (46.00–61.75)	0.211
Moderate or severe AR	33 (29.2)	33 (33.0)	0.550	18 (27.7)	20 (30.8)	0.700
Moderate or severe MS	2 (1.8)	2 (2.0)	0.902	1 (1.5)	0 (0)	0.317
Moderate or severe MR	44 (38.9)	42 (42.0)	0.649	23 (35.4)	24 (36.9)	0.855
Moderate or severe TR	25 (22.1)	21 (21.0)	0.842	15 (23.1)	16 (24.6)	0.837
MDCT information						
Systolic annular perimeter	75.40 (70.70–83.10)	79.10 (74.10–85.60)	**0**.**019**	76.90 (71.10–84.80)	79.00 (72.93–86.70)	0.300
Systolic annular area	439.80 (384.20–537.10)	482.30 (415.80–576.90)	**0**.**031**	462.50 (393.20–559.90)	484.95 (412.50–581.86)	0.316
Tricuspid aortic valve	65 (57.5)	68 (68.0)	0.115	39 (60.0)	38 (58.5)	0.858
Calcification (HU850)	435.00 (257.00–649.00)	388.00 (163.00–685.00)	0.250	468.00 (352.00–751.00)	374.00 (168.75–742.75)	0.076
Iliofemoral artery information						
Diameter	7.24 ± 1.21	6.96 ± 1.17	0.097	7.10 ± 1.18	7.30 ± 1.17	0.335
Tortuosity index	13.11 (9.13–16.44)	13.10 (9.13–18.75)	0.607	13.11 (9.21–14.08)	14.00 (9.13–18.75)	0.480
Calcification index	2.90 (0.52–6.85)	2.92 (0.58–6.62)	0.594	3.65 (0.79–7.16)	2.92 (0.64–6.62)	0.334

Values are *n* (%), mean ± standard deviation, or median with interquartile range. Boldface = significant *P* value. AR, aortic regurgitation; BMI, body mass index; BSA, body surface area; CABG, coronary artery bypass grafting; CK, chronic kidney disease; COPD, Chronic obstructive pulmonary disease; DA, dual artery access; LVEF, left ventricular ejection fraction; LVEDD, left ventricular end-diastolic dimension; LVESD, left ventricular end-systolic dimension; MDCT, multidetector computed tomography; MI, myocardial infarction; MR, mitral regurgitation; MS, mitral stenosis; NYHA, New York Heart Association; PCI, percutaneous transluminal coronary intervention; PVD, peripheral vascular disease; SA, single artery access; TR, tricuspid regurgitation; and UCG, ultrasound cardiogram.

All analyses were performed using SPSS 24.0 (SPSS Inc., Chicago, IL, USA) and Stata 14.0 (Stata, College Station, TX, USA). A two-tailed *p*-value ≤ 0.05 was considered statistically significant.

## Results

### Baseline characteristics of the total population

**Figure 2:**
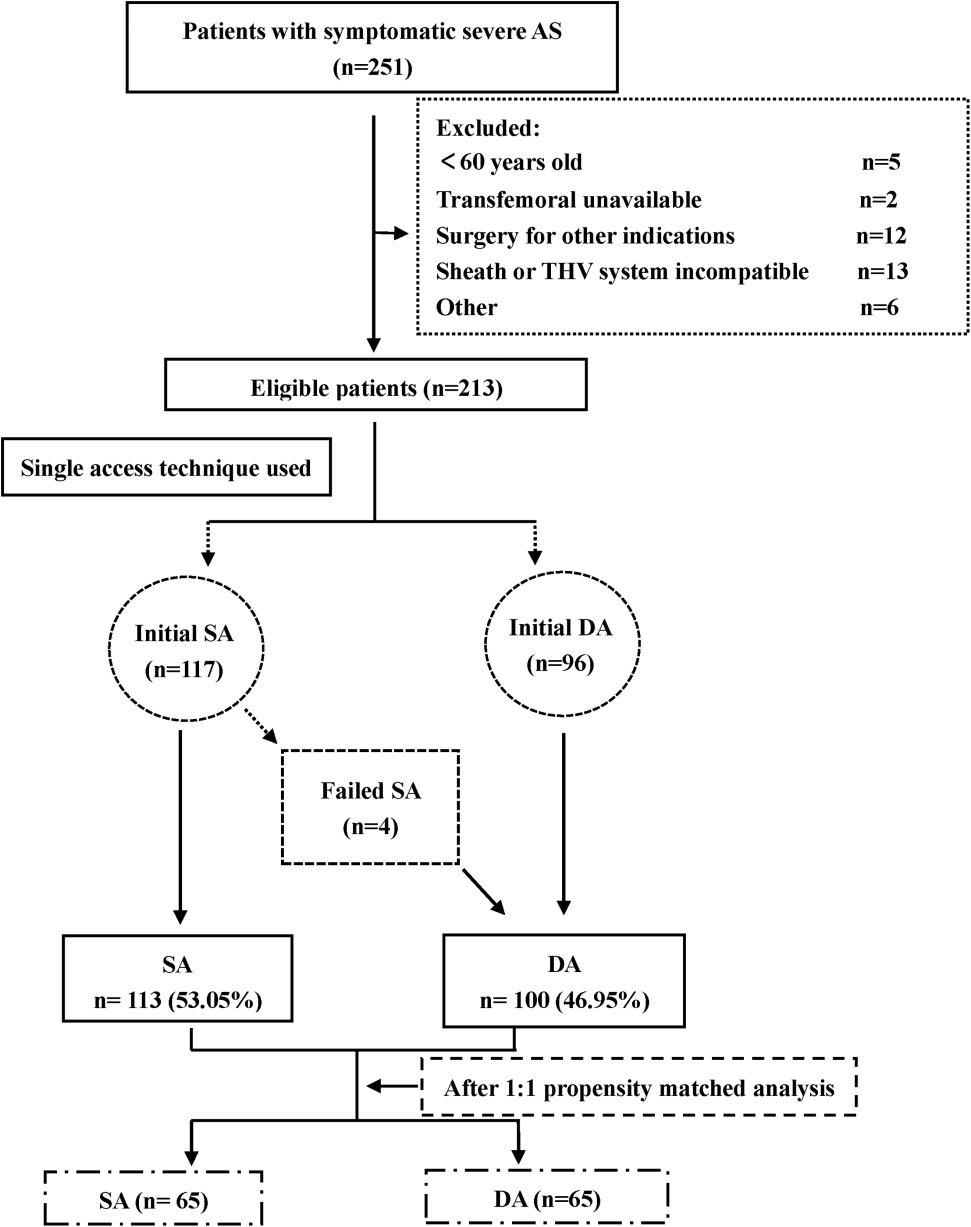
Flow chart. AS, aortic stenosis; DA, dual artery access; SA, single artery access; THV, transcatheter heart valve.

From June 2021 to September 2022, a total of 213 patients with severe AS were consecutively enrolled in the present study (Figure 2). Of these patients, 113 (53.05%) were treated by SA (the SA group), and 100 (46.95%) were treated by conventional DA (the DA group).

The baseline clinical, ultrasound cardiographic, and contrast-enhanced multidetector computerized tomography characteristics are listed in Table 1. To reiterate, patients who were treated with SA had a higher prevalence of right bundle branch block and a lower prevalence of prior stroke compared with the DA group. The perimeters of the annulus and the areas in patients with SA were statistically smaller. No significant differences existed between the groups for the other variables.

### Baseline characteristics of the propensity matched population

**Figure 3:**
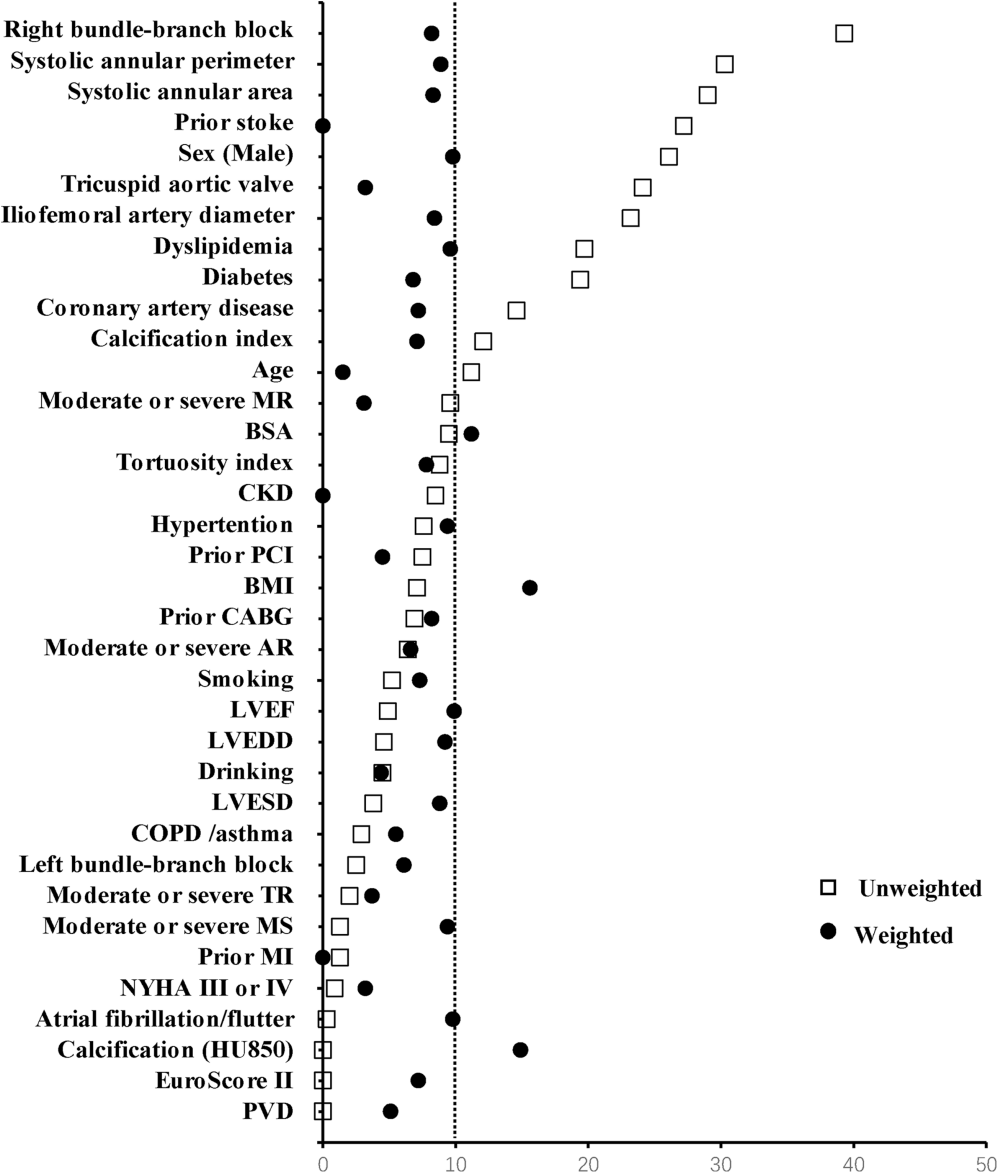
Absolute standard difference before and after propensity score matching.

After a 1:1 propensity matched analysis, 65 individuals were included in each group. The absolute standard differences after the matching were all less than 10.0% (Figure 3) except for the body surface area (BSA), body mass index (BMI) and aortic valve calcification (HU850), which showing a good matching balance. None of the baseline items showed statistical differences (Table 1).

### Procedural outcomes of the propensity matched population

Procedural outcomes are listed in Table 2. In summary, the end points for x-ray time and x-ray dose for patients in the SA group were comparable to those in the DA group, as were the end points of concurrent PCI, sheath usage, bioprosthetic valve type, sheath to femoral artery ratio (SFAR) and valve-in-valve implants. The operating times for the DA group were longer than those for the SA group (102 min vs. 125 min; *P *= 0.001).

**Table 2:** Procedural information.

	Total population (*n* = 213)			Propensity matched population (*n* = 130)		
	SA (*n* = 113)	DA (*n* = 100)	*P* Value	SA (*n* = 65)	DA (*n* = 65)	*P* Value
Operation duration (min)	105.00 (80.00–135.00)	111.00 (90.00–160.00)	**0**.**031**	**102.00** (**75.00–125.00)**	**125.00** (**90.50–160.00)**	**0**.**001**
x-ray time (min)	19.00 (14.50–25.00)	19.00 (15.00–23.44)	0.812	19.00 (15.00–25.00)	19.00 (14.70–24.50)	0.591
x-ray dose (mGy)	601.00 (403.00–1,237.00)	520.00 (411.84–850.86)	0.239	588.00 (426.00–1,112.37)	583.00 (411.88–835.81)	0.841
Concurrent PCI	16 (14.2)	15 (15.0)	0.862	8 (12.3)	13 (20.0)	0.233
VIV implant	5 (4.4)	8 (8.0)	0.277	2 (3.1)	2 (3.1)	1.000
Sheath size (Fr)						
20	101 (89.4)	86 (86.0)	0.452	57 (87.7)	56 (86.2)	0.795
22	12 (10.6)	14 (14.0)	0.452	8 (12.3)	9 (13.8)	0.795
SFAR	1.09 ± 0.23	1.11 ± 0.22	0.453	1.11 ± 0.25	1.06 ± 0.19	0.163
Bioprosthetic valve type						
Venus A/A plus	81 (71.7)	62 (62)	0.133	45 (69.2)	37 (56.9)	0.146
Taurus One/Elite	17 (15)	14 (14)	0.829	9 (13.8)	10 (15.4)	0.804
VitaFlow	15 (13.3)	24 (24)	**0**.**043**	11 (16.9)	18 (27.7)	0.140

Values are *n* (%), mean ± SD or median with interquartile range. Boldface = significant *P* value. DA, dual artery access; PCI, percutaneous transluminal coronary intervention; SA, single artery access; VIV, valve in valve; SFAR, sheath to femoral artery ratio.

### Clinical outcomes of the propensity matched population

Table 3 lists all clinical outcomes. Device success was observed in 119 individuals: 62 (95.4%) in the SA group and 57 (87.7%) in the DA group. Although the SA procedure was superior numerically, there were no statistical differences between the groups (*P *= 0.115). As to the secondary clinical end points—death; cardiac death; myocardial infarction; new permanent pacemaker and disabling stroke—those in the SA group were comparable to those in the DA group, both perioperatively and at the 1-month and 6-month follow-up examinations. There were also no statistical differences in the end points of acute kidney injury and new left bundle branch block when analyzed perioperatively. Moderate/severe paravalvular regurgitation results were also comparable between groups both perioperatively and at the 1-month follow-up, as were rehospitalizations at both the 1-month and the 6-month follow-up examinations.

**Table 3:** Clinical outcomes (total population *n* = 213).

	Total population (*n* = 213)			Propensity matched population (*n* = 130)		
	SA (*n* = 113)	DA (*n* = 100)	*P* Value	SA (*n* = 65)	DA (*n* = 65)	*P* Value
Perioperatively						
Main vascular/access complication						
Major	0 (0.0)	0 (0.0)	NA	0 (0.0)	0 (0.0)	NA
minor	1 (0.9)	2 (2.0)	0.488	0 (0.0)	2 (3.1)	0.156
Secondary vascular/access complications						
major	0 (0.0)	0 (0.0)	NA	0 (0.0)	0 (0.0)	NA
minor	0 (0.0)	0 (0.0)	NA	0 (0.0)	0 (0.0)	NA
Deaths	1 (0.9)	2 (2.0)	0.492	0 (0.0)	0 (0.0)	NA
Cardiac deaths	1 (0.9)	1 (1.0)	0.931	0 (0.0)	0 (0.0)	NA
Myocardial infarction	0 (0.0)	0 (0.0)	NA	0 (0.0)	0 (0.0)	NA
AKI	0 (0.0)	0 (0.0)	NA	0 (0.0)	0 (0.0)	NA
Moderate/severe paravalvular regurgitation	1 (0.9)	5 (5.0)	0.071	1 (1.5)	2 (3.1)	0.561
New LBBB	19 (16.8)	25 (25.0)	0.141	9 (13.8)	15 (23.1)	0.175
New PPM	14 (12.4)	8 (8.0)	0.293	4 (6.2)	5 (7.7)	0.730
Disabling stroke	1 (0.9)	0 (0.0)	0.347	0 (0.0)	0 (0.0)	NA
1-Month follow-up						
Device success	105 (92.9)	85 (85.0)	0.063	62 (95.4)	57 (87.7)	0.115
Deaths	1 (0.9)	3 (3.0)	0.258	0 (0.0)	1 (1.5)	0.317
Cardiac deaths	1 (0.9)	2 (2.0)	0.492	0 (0.0)	1 (1.5)	0.317
Myocardial infarction	0 (0.0)	0 (0.0)	NA	0 (0.0)	0 (0.0)	NA
Rehospitalization	10 (8.8)	3 (3.0)	0.076	6 (9.2)	1 (1.5)	0.053
Moderate/severe paravalvular regurgitation	1 (0.9)	5 (5.0)	0.071	1 (1.5)	2 (3.1)	0.561
New PPM	21 (18.6)	10 (10.0)	0.077	7 (10.8)	6 (9.2)	0.770
Disabling stroke	1 (0.9)	0 (0)	0.347	0 (0.0)	0 (0.0)	NA
6-Month follow-up						
Deaths	1 (0.9)	3 (3.0)	0.258	0 (0.0)	1 (1.5)	0.317
Cardiac deaths	1 (0.9)	2 (2.0)	0.492	0 (0.0)	1 (1.5)	0.317
Myocardial infarction	0 (0.0)	0 (0.0)	NA	0 (0.0)	0 (0.0)	NA
Rehospitalization	10 (8.8)	3 (3.0)	0.076	6 (9.2)	1 (1.5)	0.053
New PPM	22 (19.5)	10 (10.0)	0.054	8 (12.3)	6 (9.2)	0.571
Disabling stroke	1 (0.9)	0 (0)	0.347	0 (0.0)	0 (0.0)	NA

AKI, acute kidney injury; DA, dual artery access; LBBB, left bundle-branch block; PPM, permanent pacemaker; SA, single artery access.

### Vascular/access complication of the propensity matched population

Only two vascular/access complications occurred. All those complications were in the DA group and minor main vascular/access events. There was no statistical significance between groups (0.0% vs. 3.1%, *P *= 0.156) (Table 3).

## Discussion

We analyzed a modified minimally invasive TAVR procedure in which only one artery was punctured (without upgrading the sheath size). To the best of our knowledge, this is a most innovative attempt. After comparing the outcomes of our procedure with those of the DA procedure and applying the method of propensity matched analysis, our main findings are as follows: (1) The SA procedure achieved similar device success compared with the DA procedure. (2) The SA procedure shortened the operating time and did not increase the x-ray time or dose. (3) The 20 Fr and 22 Fr sheaths could be used for the SA procedure with a low incidence of vascular/access complications that was comparable to the results achieved with the DA procedure.

Previous studies have contributed useful suggestions for simplifying the TAVR approach. Secondary access was the primary focus. The radial artery was a reasonable alternative. Curran and colleagues enrolled 87 patients (11) and by comparing the radial artery access with the collateral femoral access, they proved the safety and efficiency of the radial access (major vascular complication: 4.3% vs. 7.3%, *P *= 0.553). Other researchers reported similar results (9, 17, 18). Khubber and colleagues reported their experience with the unilateral access technique (13). They punctured both the main and secondary access points in the same femoral artery. A significant decline (6.3%) in the incidence of vascular complications was observed after they applied the unilateral access technique. However, vascular complications related to secondary access still occurred. Gianluca and colleagues published a case report in which they did not mark a secondary access point (19). With the help of visible aortic root calcification, a prosthetic aortic valve was successfully implanted. Nicholas and colleagues also reported an analysis of such SA techniques (14). A total of 100 patients were enrolled consecutively. When compared with conventional DA, the SA technique showed a high incidence of technical success (100% vs. 100%, *P *= 1.000) and a low incidence of complications. However, they used anatomical calcification as a landmark to obviate the need for a secondary access. This SA technique requires a rigorous protocol for patient selection and is difficult to extrapolate to all patients with AS. Stefan and colleagues shared their experience with a modified SA TAVR procedure (15): By placing both the delivery system and the pigtail into the sheath, they achieved promising procedural and clinical outcomes. However, they reported only the results of a single arm study and did not compare their results with those from the DA procedure. We reported our experience with a modified SA procedure. When we compared the results of the modified SA procedure with those of the DA procedure, we found that the SA procedure achieved similar device success. The operating time was shorter. At the same time, it did not increase the x-ray time or dose. We believe that such an SA technique is promising and can be extrapolated to all TAVR procedures. Larger randomized controlled trials are needed to further prove the safety and efficiency of the SA technique.

The incidence of major/minor vascular/access-related complications in this study was low. Previous studies and all-comer cohort studies reported a vascular complication incidence of approximately 10%-20% (6, 20–22). More recent reports (mainly RCTs) have shown an incidence of 4% or less (23–26). Possible explanations for these results are as follows: (1) Real-time ultrasound-guided needle puncture helps interventionists clearly distinguish calcifications, the anterior wall of an artery, the branch artery, and the vein. Previous studies have proven that, compared to fluoroscopy guidance and anatomical landmark guidance, ultrasound guidance during femoral catheterization can significantly reduce the incidence of access-related complications (27–29). In our centre, when we evaluated an approach using contrast-enhanced multidetector computerized tomography, the distance between the femoral bifurcation and the ideal femoral artery puncture site was premeasured. Thus, interventionists were more likely to achieve a precise puncture, which might help keep vascular complications to a minimum. (2) We performed a diligent preprocedural evaluation of the vascular access. (3) We used a percutaneous closure device. In the present study, two ProGlide closure devices were used to suture the artery. The haemostatic efficiency of this technique has been reported by previous researchers (30, 31). (4) We had a small sample of only 213 patients. The small sample size might also explain the lower incidence.

We reported herein our modification of the SA technique. Some key techniques should be highlighted: (1) As described previously, ultrasound-guided puncture contributed to a decrease in the incidence of vascular complications. (2) The entry and exit of the 5 Fr pigtail catheter should be repeated to ensure the trafficability of the devices. (3) We introduced another new technique in which contrast agent was slowly injected while the sheath was removed to avoid causing a vascular injury. Traditionally, angiography was performed from the secondary approach to detect the potential for vascular injury even though, to our knowledge, this manual injection technique is safe and efficient. Further studies are needed to prove this hypothesis.

## Limitations

The present study has some limitations: (1) Because the sample size is small, it is difficult to draw definitive conclusions. Further studies with a large sample are needed to verify the feasibility of the modified SA technique. (2) Some sheath or THV delivery systems could not be used to perform the SA technique. The construction of the e-sheath (Edwards Lifesciences) did not permit the insertion of 2 instruments. Thus, the SAPIEN (Edwards Lifesciences) valves were excluded from the present study. Further studies are needed to confirm that the SAPIEN is suitable for the modified SA technique. This study was a single-center, retrospective study, although we did perform propensity score matched analyses. Our evidence grade was indeed lower than that of multicenter randomized controlled trials. (4) Patients with aortic regurgitation were not enrolled in the present study.

## Conclusions

The SA procedure is a promising minimally invasive TAVR technique with a low incidence of vascular complications and a high incidence of device success. Such an innovative technique is safe and might be suitable for application in all TAVR procedures.

## Data Availability

The raw data supporting the conclusions of this article will be made available by the authors, without undue reservation.
